# SARS-CoV-2 spike protein causes synaptic dysfunction and p-tau and α-synuclein aggregation leading cognitive impairment: The protective role of metformin

**DOI:** 10.1371/journal.pone.0336015

**Published:** 2025-11-07

**Authors:** Hye-Kyung Lee, Ji Young Choi, Jung Hyun Park, Moon Han Chang, Jung Ho Park, Young Ho Koh

**Affiliations:** 1 Division of Brain Diseases Research, Department of Chronic Disease Convergence Research, Korea National Institute of Health, Cheongju-si, Republic of Korea; 2 Nexmos Inc., Yongin-si, Gyeonggi-do, Republic of Korea; The University of Texas Rio Grande Valley School of Medicine, UNITED STATES OF AMERICA

## Abstract

Patients recovering from post-coronavirus disease 2019 (COVID-19) often experience cognitive dysfunction, including difficulties with focusing, conversation, or memory issues, which can persist for weeks or even months after infection. Although the persistence of the SARS-CoV-2 spike protein has been reported in both the brain and serum, and is thought to potentially contribute to the long-term effects of SARS-CoV-2 infection on the brain, the underlying mechanisms leading to cognitive dysfunction remain unclear. This study investigates the molecular mechanisms by which the SARS-CoV-2 S1 protein (hereafter S1) induces cognitive impairment and explores the therapeutic potential of metformin in mitigating these effects. We demonstrate that intranasally administered S1 quickly entered the hippocampus and was associated with cognitive impairment by 6 weeks post-injection. Transcriptomic analysis of hippocampal tissue revealed early alterations in gene expression associated with synaptic function. We observed that the expression of hypoxia-responsive genes was also altered, suggesting the involvement of HIF-1α signaling. Further analysis confirmed that S1 stabilized the HIF-1α protein in a hypoxia-independent manner, and siRNA-mediated knockdown of HIF-1α restored synaptic gene expression, including GRIN2A, SHANK1, and JPH3. By 6 weeks post-injection, hippocampal neuronal loss was accompanied by the accumulation of phosphorylated tau (p-tau) and aggregated α-synuclein. Notably, treatment with metformin rescued synaptic gene expression and attenuated p-tau and α-synuclein aggregation. These findings suggest that S1 disrupts synaptic homeostasis and promotes neurodegenerative processes, and that metformin may serve as a potential therapeutic strategy to mitigate long-term cognitive sequelae of COVID-19.

## Introduction

Over 776 million confirmed cases of COVID-19 have been reported worldwide [1]. Despite recovering from the acute phase of the disease, some COVID-19 survivors experience persistent symptoms or other health issues lasting for weeks to months, and in some cases even years after post-infection. This condition, known as long COVID or postacute sequelae of SARS-CoV-2 infection (PASC), has been widely documented [2,3]. The symptoms of long COVID affect multiple organ systems and include loss or alteration of smell or taste, postexertional malaise, chronic cough, fatigue, brain fog, and more [4]. Brain fog, in particular, is characterized by difficulties in concentration, memory, conversational tracking, and slowed processing speed. Several large-scale cohort and meta-analytic studies further indicate that these cognitive deficits persist in a significant subset of survivors for extended periods. For instance, a systematic review reported a pooled prevalence of post-COVID cognitive dysfunction of approximately 22% beyond 12 weeks after infection, with symptoms such as memory loss and concentration difficulties persisting up to one year or longer in many cases [5,6]. Approximately 28% of COVID-19 survivors report experiencing brain fog, with the relative risk of mental health and neurological issues—such as adjustment disorders or memory problems—remaining significantly elevated for up to two years after infection [7,8]. Beyond impairing quality of life, these sequelae may predispose vulnerable individuals to neurodegenerative processes, including an increased risk of dementia. Recent large-scale cohort analyses have reported that COVID-19 survivors exhibit a higher incidence of new-onset dementia compared to matched controls, particularly in older adults and those with severe infections [5,9]. Consequently, increasing efforts have focused on elucidating the mechanisms by which SARS-CoV-2 contributes to long-term neurological complications.

To explain the long-term effects of SARS-CoV-2 infection on the brain, researchers have suggested the possibility of the virus invading the brain; SARS-CoV-2 infection in the nasal cavity may spread to the brain via the olfactory nerves, and it is evidenced by the detection of SARS-CoV-2 and its viral proteins in the skull, meninges, and brain [10,11]. The SARS-CoV-2 spike protein has been shown to persist in immune cells for at least 15 months and in blood plasma for at least 12 months in individuals who were infected with the virus [12,13]. A clinical study found that long COVID is associated with persistent residual SARS-CoV-2 antigen including the S1 subunit of the spike, full-length spike, and nucleocapsid [13]. Additionally, injection of the SARS-CoV-2 S1 protein (hereafter referred to as S1), the surface domain of SARS-CoV-2 responsible for binding to the human angiotensin-converting enzyme 2 (ACE2) receptor, into the mouse hippocampus induced neuroinflammation and microglial gliosis, leading to cognitive dysfunction [14]. Overall evidence suggests that the S1 is pivotal in COVID-19 pathogenesis. While inflammation and reactive oxygen species (ROS) triggered by SARS-CoV-2 are thought to be key factors in its pathogenesis, more research is needed to understand how the presence of SARS-CoV-2 spike protein in the brain progressively leads to neurological disorders, including cognitive impairment, in post-COVID patients.

Previously, viral infections such as human immunodeficiency virus, West Nile virus, H1N1 influenza A virus, or herpes simplex virus type 1 caused several neurological and psychiatric illnesses. For example, the spike protein of herpes simplex virus type 1 (HSV-1) binds to heparin and increases aggregation of amyloid beta (Aβ) leading to amyloidosis associated with AD [15]. A recent study suggests that the S1 binds heparin and heparin-binding proteins, which are prone to self-assembly and aggregation processes that could contribute to post-COVID-19 complications including neurodegeneration [16]. *Idrees & Kumar* reported that the S1 can trigger the aggregation of neuropathogenic proteins including Aβ, tau, and α-synuclein causing the development and progression of neurodegenerative diseases, such as AD and PD [16]. Particularly, α-synuclein aggregates are a primary pathological feature of diverse neurodegenerative diseases including PD, MSA, and motor movement disorders, which are termed α-synucleinopathies [17]. Our previous study showed that the intranasal administration of S1 to mice increases aggregation of α-synuclein through brain inflammation in the substantia nigra and a dopaminergic cell line [18]. These α-synuclein aggregates are co-localized with hyperphosphorylated tau form, neurofilamentary tangle, and Aβ plaque in the postmortem brain with AD. From *in-vitro* and *in-vivo* studies, it is confirmed that the interaction of α-synuclein and phosphorylated tau (p-tau) promotes their fibrillization, which causes neurodegeneration. Therefore, the S1 forms toxic aggregates that can act as seeds to aggregate many of the misfolded proteins and progress neurological disorders.

The current study investigates the molecular mechanism underlying S1-induced cognitive impairment. We confirmed that cognitive dysfunction was induced following S1 administration and analyzed the genes altered in the hippocampus. Within 1 week of S1 administration, the expression of genes associated with synaptic function was downregulated. Furthermore, we found that HIF-1α, stabilized by S1 protein independently of hypoxic conditions, regulates the expression of synaptic genes. At 6 weeks post-administration, hippocampal cell death was accompanied by the accumulation of neuropathogenic proteins, including p-tau and aggregated α-synuclein. Metformin reversibly inhibited S1-induced HIF-1α activation and the aggregation of neuropathogenic factors. Therefore, the purpose of this study was to elucidate the molecular mechanisms by which S1 induces cognitive impairment and to explore the potential of metformin as a therapeutic agent.

## Materials and methods

### Reagents

Recombinant SARS-CoV-2 S1 spike protein (His-tagged) was sourced from R&D Systems (Minneapolis, MN; #10522-CV), and metformin hydrochloride was obtained from Sigma-Aldrich (Burlington, MA; #PHR1084).

### In vivo administration of S1 protein

Male Sprague-Dawley rats were maintained under standard light-dark cycles with free access to food and water. All experimental procedures complied with NIH guidelines (2013) and ARRIVE recommendations, and were approved by the KCDC Institutional Animal Care and Use Committee (KCDC-IACUC-22-008). Anesthesia was achieved using intramuscular ketamine/xylazine (2:1 ratio, 100–150 μL/100 g). Animals were randomly assigned to receive either S1 (n = 12) or phosphate-buffered saline (PBS; n = 12) via intranasal delivery. The procedure, adapted from Thorne et al. (2004) [19], involved administering 10 μL of S1 suspension (0.5 μg) into alternating nostrils of supine rats using a 26-G Hamilton microsyringe at 2-minute intervals.

### Behavioral testing

Behavioral experiments were conducted at three-day intervals following treatment. Each animal underwent a battery of tests in the following sequence: open field, episodic-like memory, and Morris water maze. All tests were performed in a quiet, sound-attenuated room after at least 30 min of acclimation. Animal movements and behavior were recorded and analyzed using a video tracking system (Panlab, Barcelona, Spain).

#### Open field test.

This assay evaluated general locomotion and anxiety-like behavior in an 80 × 80 × 50 cm square arena under low lighting. Rats were allowed to explore freely for 10 min, and parameters such as distance traveled and time spent in center versus periphery were quantified.

#### Episodic-like memory test.

**Fig 1:**
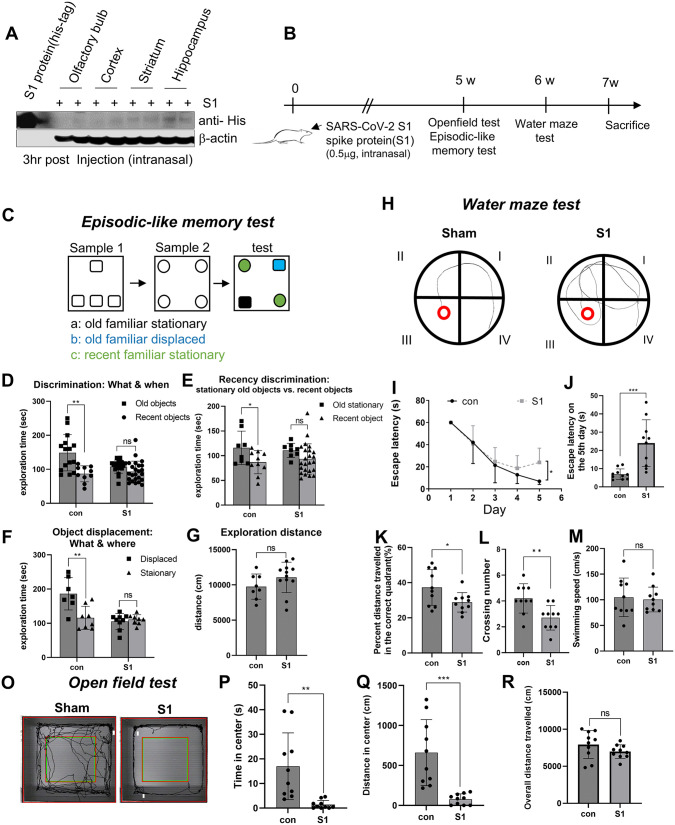
S1 invasion to the brain and its effects on learning and memory. (A) Intranasally injected S1 (His-tagged) in the olfactory bulb, striatum, and hippocampus was examined at 3 hr post S1 administration (0.5 μg/per animal) using immunoblotting. Representative images are presented. (B) Schematic of behavior tests. (C-G) Episodic-like memory task. (C) The episodic-like memory task consists of two sample phases and a test phase. In each sample phase, rats encountered two sets of four identical novel objects (old objects, sample 1; recent objects, sample 2). In the test phase, objects were mixed together. One of the old familiar objects was placed in new location (b: old familiar displaced). The other three objects were placed in same location as in sample phases (a: old familiar stationary, c: recent familiar stationary). (D) Distribution of exploratory time for ‘what-when’ (old familiar objects vs. recent familiar objects). (E) Recency discrimination of exploratory times for ‘what-when’ (old stationary objects vs. recent stationary objects) (F) Distribution of exploratory time for ‘what-where’ (old familiar displaced vs. recent familiar stationary). (G) Total exploratory distance for each group in the test phase. (H-M) Water maze test. (H) Representative swimming paths of the control and S1 injected group during the probe trial. (I-J) The escape latencies in the two groups over five consecutive training days (I) and on the 5th day of the spatial acquisition session (J). (K-L) The percentage of time spent and distance traveled in the target quadrant during the probe trial (K), the average crossing number over the platform-site, and the latency of the first target-site crossover (L). (M) The average swimming speed of two groups. (O-R) Open field test. (O) Representative track sheets of the control and S1 injected group during the test trial. (P-R) Graphs showing alterations in time spent in central zone (P), distance traveled in central zone (Q), and overall distance traveled (R). Values are presented as means±SEMs. * p < 0.05, ** p < 0.01, ***p < 0.001 vs. control. Con, control group (n = 12); S1, S1 injected group (n = 12).

This task was designed to evaluate components of episodic memory, including the “what”, “where”, and “when” aspects. Behavioral testing was performed according to a previously established protocol with minor modifications [20]. In brief, animals were first handled individually for 7 days, then habituated to the open field for 3 additional days (5 min/day) without objects. Object familiarization involved two daily sessions (10 min per session, 20 min interval) over two days with two identical objects in opposing corners. Testing involved two sample trials and one test trial. On the first sample trial, the rats were placed in the center of the open field containing four novel objects arranged in a rectangular configuration (as illustrated in Fig 1C) and allowed to explore them for 10 min. After 50 min inter-trial interval, the rats received the second sample trial that 4 novel objects were placed in the corners of the open field (Fig 1C). After a delay of 50 min, the rats received a test trial that two copies of the object from sample trial 1 (“old familiar” objects placed in the north-east and south west) and two copies of the object from sample trial 2 (“recent familiar” objects placed in the north-west and south-east) were present (Fig 1C).

#### Morris water maze test.

Spatial learning and memory were evaluated using the Morris water maze, as described in previous studies [21]. Sham control and S1 protein-injected rats were trained in a circular pool (diameter: 180 cm, depth: 30 cm) filled with opaque water maintained at 22 ± 1°C. The maze was divided into four quadrants (I-IV), with a hidden escape platform (15.2 cm in diameter) placed in quadrant III (target quadrant), submerged approximately 1 cm below the water surface. A video camera (Sony, Tokyo, Japan) mounted above the pool recorded the rats’ movement. Each rat received one trial per day over five consecutive days for spatial acquisition training. Rats were released from a random start location and given 60 s to locate the hidden platform. If unsuccessful, the rat was gently guided to the platform. After the training phase, a probe trial was conducted without the platform to assess spatial memory retention. Parameters analyzed included escape latency, first crossing latency, number of crossings over the platform site, and time and distance spent in the target quadrant. Data were analyzed using the Panlab video tracking system.

### mRNA sequencing

1 week after intranasal administration of the S1 protein, rats were sacrificed and hippocampal tissues were rapidly harvested. Total RNA was extracted using the RNeasy Mini Kit (Qiagen, Germany) per the manufacturer’s instructions. Sequencing was performed commercially (EBIOGEN, Seoul, Korea) using the HiSeq X10 platform to generate 4G paired-end reads. Raw data were processed with BBDuk, and reads were aligned to the rn6 reference genome using TopHat. Gene-level counts were computed, and differentially expressed genes (DEGs) were identified using ExDEGA (fold change ≥ |1.6|, p ≤ 0.05).

### Functional annotation and pathway analysis

Gene ontology (GO) and pathway enrichment analyses were conducted using the Database for Annotation, Visualization and Integrated Discovery (DAVID, https://david.ncifcrf.gov/home.jsp, Version 6.8). Genes were annotated based on rattus norvegicus gene symbols, and significant terms (p < 0.05) were reported for biological process, molecular function, and cellular component categories.

### Gene-set enrichment analysis (GSEA)

All detected genes, regardless of differential expression, were included in a GSEA using GO Biological Process gene sets (http://software.broadinstitute.org/gsea/index.jsp). For preranked GSEA anlaysis, signed −log10 FDRs from DESeq2 analyses were used via fgsea v1.16.0, filtering out genes with an FDR > 0.5. Public gene sets, GO Biological Processes (GO db v2024) used for analyses.

### Immunohistochemistry

Following fixation in 4% paraformaldehyde and cryoprotection in 30% sucrose, brains were embedded in paraffin and sectioned at 5 µm. Sections were deparaffinized, rehydrated, and blocked with a solution containing 5% FBS, 5% horse serum, 2% albumin, and 0.1% Triton X-100 [22]. Primary antibodies targeting NeuN (Millipore, Burlington, MA), HIF-1α (BD Biosciences, Bedford, MA), NMDAR2A (Cell signaling technology, Danvers, MA), JPH3 (Invitrogen, Waltham, MA), phospho Tau (T205) (Abcam, Cambridge, UK), or aggregated α-synuclein (Merk Millipore, Darmstadt, Germany) were used at a concentration of 1:100. After incubation with primary antibodies, brain sections were washed with PBS and incubated with fluoresence labeled anti-rabbit IgG secondary antibody (1:200; Jackson ImmunoRes, West Grove, PA). Sections were counterstained with DAPI (Vector Laboratories, Peterborough, UK) and coverslipped.

### Cell culture and siRNA treatment

N2A and H4 neuronal cell lines were maintained in DMEM supplemented with 10% FBS and 1% penicillin-streptomycin at 37°C in 5% CO₂. For in vitro HIF-1α stabilization assays, cells were treated with recombinant S1 (0.5 μg/mL) for up to 24 hr. For gene silencing experiments, siRNA targeting HIF-1α (siHIF-1α; Dharmacon, Lafayette, CO) was transfected using Lipofectamine 3000 (Invitrogen, Thermo Fisher Scientific, Waltham, MA) according to the manufacturer’s protocol. H4 cells stably overexpressing SUMO1, named HGS1 (OriGene Technologies Inc., Rockville, MD), were used to examine SUMO1-mediated effects on HIF-1α stabilization.

### TUNEL assay

Apoptotic cell death was detected using the In Situ Cell Death Detection Kit, Fluorescein (Roche, Basel, Switzerland), following the manufacturer’s instructions. Brain sections were counterstained with DAPI and examined using fluorescence microscopy.

### Immunoblot analysis

Brain homogenates or whole-cell lysates were extracted with RIPA buffer (Thermo Fisher Scientific, Waltham, MA) quantified via BCA assay, and resolved by SDS-PAGE (8–14%)(Thermo Fisher Scientific, Waltham, MA). Western blots were probed with primary antibodies against anti-His (1:1,000; Thermo Fisher Scientific, Waltham, MA), anti-HIF-1α (1:1,000; Cell Signaling Technology, Danvers, MA), anti-NMDAR2A (1:1,000; Cell signaling technology, Danvers, MA), anti-JPH3 (1:1,000; Thermo Fisher Scientific, Waltham, MA), anti-phospho-Tau (Ser202, Thr205) (AT8) (1:1,000; Thermo fisher scientific, Waltham, MA), anti-Tau (Tau-5)(1:1,000; Thermo fisher scientific, Waltham, MA), anti-aggregated α-synuclein (1:1,000; Merk Millipore, Darmstadt, Germany), anti-SUMO1(1:1,000; Thermo fisher scientific, Waltham, MA) or anti-β-actin (1:5,000; Thermo Fisher Scientific, Waltham, MA) antibody. Blots were detected using anti-rabbit HRP-conjugated anti-rabbit or anti-mouse HRP secondary antibody (1:2,000, MilliporeSigma, Burlington, MA) and a chemiluminescence kit (Thermo Fisher Scientiﬁc, Waltham, MA).

### RNA preparation and realtime PCR

RNA was prepared using TRIzol reagent (Thermo Fisher Scientiﬁc, Waltham, MA) and reverse-transcribed. PCR amplification was carried out on a QuantStudio 3 system using gene-specific primers for mouse Glutamate Ionotropic Receptor NMDA Type Subunit 2A (GRIN2A), mouse Glutamate Ionotropic Receptor AMPA Type Subunit 2 (GRIA2), mouse SH3 and Multiple Ankyrin Repeat Domains 1 (SHANK1), mouse Junctophilin (JPH3), and GAPDH were as follows: GRIN2A, 5’- CGT AGA GGA TGC CTT GGT CA −3’ (sense) and 5’ – CCA TAG CCT GTG GTG GCA AA −3’ (antisense); JPH3, 5’ – CGG AGC CAA ATA CGA AGG GA −3’ (sense) and 5’- CCA TAA GGG ACA CTC TGC CG −3’ (antisense); SHANK1, 5’- CTC CTC TCC AAA CCC AGC AG −3’ (sense) and 5’- GGC CGC ATC TCA AAT TCC AC −3’ (antisense); GRIA2, 5’-CCC ATC GAA AGT GCT GAG GA −3’ (sense) and 5’- CCT CAC AAA CAC AGA GGG CT −3’ (antisense). Reactions were run in triplicate using the Premix Ex Taq system (Takara Bio Inc., Shiga, Japan), and relative expression was analyzed using the ΔΔCt method.

### Statistical analysis

Data were analyzed using GraphPad Prism 7.0 (GraphPad Software Inc., San Diego, CA). Unpaired two-tailed student’s t-tests were used for two-group comparisons, while one-way or two-way ANOVA with post hoc tests were used for multi-group analyses. Results are presented as mean ± SEM, with statistical significance set at p < 0.05.

## Result

### Intranasally administered SARS-CoV-2 spike protein contributes to learning and memory impairment

The persistent presence of SARS-CoV-2 antigens, including the spike and nucleocapsid proteins, in 65% of long COVID patients may contribute to neurological and psychiatric symptoms [13], as shown by spike protein injection into the mouse hippocampus [23]. To investigate whether S1 affects learning and memory, we intranasally administered S1 to rats. Based on previous evidence that intranasally delivered proteins can enter the brain through the olfactory route [24,25], we examined brain regions 3 hr after administration. This transport is thought to occur through cerebrospinal fluid (CSF) bulk flow along perivascular spaces that connect the nasal epithelium, olfactory nerves, and deeper brain regions, and are subsequently cleared along venous perivascular pathways [24,25]. Western blot analysis revealed that S1 protein was clearly detected in the hippocampus and striatum, indicating that the protein had penetrated deep brain regions (Fig 1A). The absence of S1 signal in the olfactory bulb at this time point may reflect a rapid peak and subsequent decline in protein concentration within this region following intranasal delivery, likely due to perivascular CSF transport and clearance through interconnected arterial and venous pathways, as previously reported [24,25].

To assess episodic-like memory, we performed a single-trial object exploration task 6 weeks after S1 injection, evaluating the binding of what-where-when components (Fig 1C). During the task, animals encountered two groups of objects at different times and in different locations (Fig 1C). The control group (PBS-injected) exhibited a consistent exploratory pattern as previously described [20]. In control rats (n = 12), exploration time was significantly greater for old familiar objects (a and b) compared to recent familiar objects (c) (Fig 1D, p = 0.0038, unpaired two-tailed Student’s t-test). To exclude the influence of object displacement on exploration preference, we compared stationary old familiar objects (a) with recent familiar objects (c); control animals still spent significantly more time exploring the old stationary objects (Fig 1E, p = 0.047). Furthermore, control rats exhibited a preference for the displaced old familiar object (b) over the stationary old familiar object (a), indicating sensitivity to spatial changes (Fig 1F, p = 0.0052). In contrast, S1-injected rats (n = 12) showed no clear preference for object exploration (Fig 1D–1F), despite no significant difference in total exploration distance between groups (Fig 1G). This confirms that S1-injected rats were not able to discriminate the relative recency of two familiar objects or detect a spatial displacement, indicating impairment in the integration of “what–when” and “what–where” memory components compared to the control group.

In the Morris water maze test, S1-injected rats exhibited random search patterns, in contrast to the goal-directed trajectories of control rats, despite comparable escape latency during spatial acquisition (Fig 1H). In the control rats (n = 12), escape latency progressively decreased over five training days, reaching 6.9 ± 0.9 s on day 5 (11.6% of day 1 latency; Fig 1I). In contrast, S1-injected rats required significantly more time (24.0 ± 4.1 s) to find the platform on day 5, approximately 3.5-fold longer than controls (Fig 1I and 1J; p = 0.0007). In the probe test, S1-injected rats showed a significant reduction in both the time spent and distance traveled in the target quadrant compared to controls (by 56%; p = 0.0322; Fig 1K). The platform crossing number was also markedly lower in the S1 group (2.7 ± 0.3 vs. 5.0 ± 0.4 in controls; p = 0.005; Fig 1L), without differences in swimming speed between groups (Fig 1M).

In addition, in the open field test, the S1-injected group spent significantly less time in the center (Fig 1P; p = 0.002), traveled less distance within the center than control group (Fig 1Q; p = 0.0003). There were no significant differences in the overall distance traveled by two groups (Fig 1R). These results indicate that S1 leads to learning and reference memory impairment, along with increased anxiety, 6 weeks after administration.

### Intranasally administered SARS-CoV-2 spike protein alters the expression of genes involved in hypoxia, neuronal injury, and synaptic plasticity

**Fig 2:**
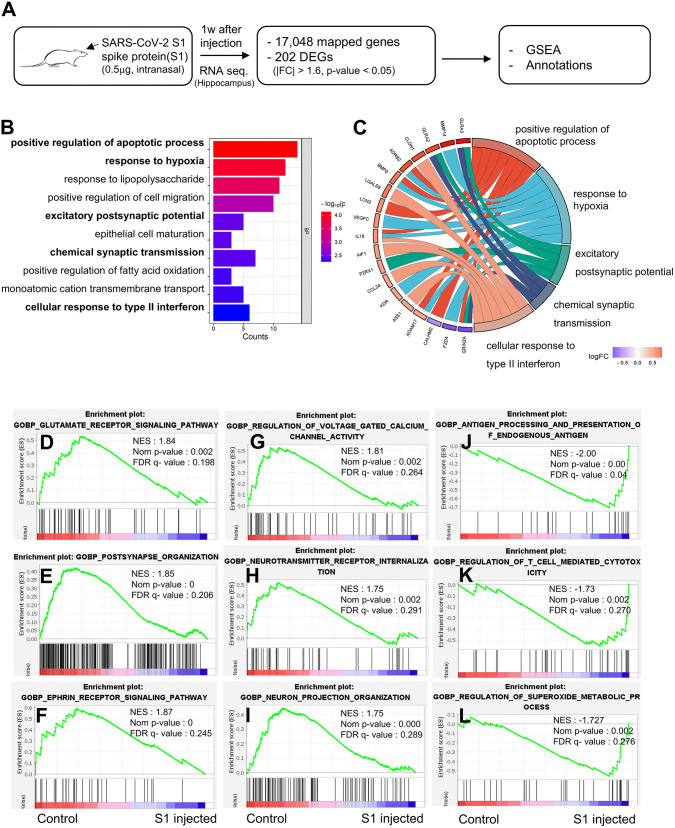
S1-mediated transcriptome changes associated with synapse activity, immune response or ROS regulation. (A) Schematic of gene expression profiling in animals injected with S1 (0.5 μg/ per animal, intranasal). (B) Gene ontology (GO) analysis of differentially expressed genes (DEGs) between control and S1 injected group showing the top enriched pathways of DEGs. (C) Chord plot showing the overlapped genes among positive regulation of apoptotic process (GO:0043065), response to hypoxia (GO:0001666), excitatory postsynaptic potential (GO:0060079), chemical synaptic transmission (GO:0007268), cellular response to type II interferon (GO:0071346). Red indicates upregulated and blue for downregulated. (D-L) Gene Set Enrichment Analysis (GSEA) for the samples of control vs. S1 injected group revealed that: genes involved in synapse activity (D-I) that were downregulated, and genes involved in immune response (J-K) and ROS regulation (L) were upregulated in the S1 injected group. NES (normalized enriched score), p-value, and FDR q-value are indicated.

To investigate the mechanism underlying S1 protein-induced learning and reference memory loss, we analyzed gene expression changes in the hippocampus 1 week after administration. Differentially expressed genes (DEGs) were identified and analyzed using Gene Ontology (GO) terms and Gene Set Enrichment Analysis (GSEA) (Fig 2A). Based on the thresholds (|Fold change| > 1.6; p < 0.05), we identified 202 DEGs and determined the functional roles of these DEGs using DAVID Bioinformatics Resources [26] (Fig 2B). GO analysis revealed enrichment in processes such as apoptosis, hypoxia response, excitatory postsynaptic potential, chemical synaptic transmission, and immune responses (Fig 2C). Notably, S1 treatment was associated with reduced synaptic plasticity and increased apoptotic signaling. GSEA further confirmed negative enrichment of synaptic activity and the positive enrichment of immune and oxidative stress pathways (Fig 2D–2L and S1 Fig).

Moreover, transcriptomic comparisons revealed that S1-injected rats and severe COVID-19 patients shared enriched GO terms related to apoptosis, synaptic transmission, and hypoxia response (S2 Fig). DEG analysis for cognition-related genes (GO:0050890) identified 251 overlapping genes, including GRIN2A and SHANK1 (S2 Fig and S1 Table). Additionally, up- and downregulated genes in the S1 model showed consistent expression patterns with those in COVID-19 patients (S2 Fig). These findings suggest that S1 disrupts hippocampal gene expression linked to hypoxia, neuronal injury, and synaptic plasticity, contributing to cognitive deficits.

**Fig 3:**
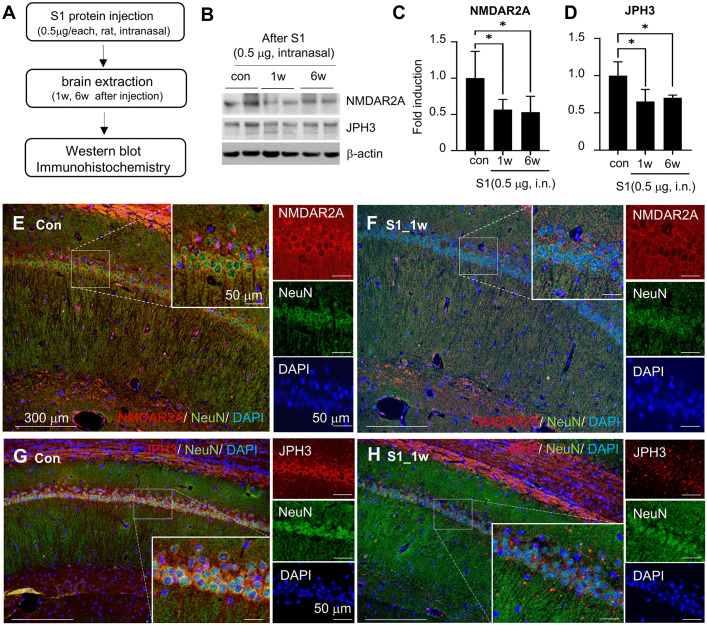
S1-mediated expression change of NMDAR2A and JPH3 in the hippocampus. (A) Schematic of immunohistochemistry and western blot in animals injected with S1 (0.5 μg/per animal, intranasal) (B-D) Hippocampus tissues were obtained at 1 or 6 weeks after S1 injection (0.5 μg/ animal, intranasal). The protein levels of NMDAR2A and JPH3 were measured by immunoblotting (B) and protein levels of NMDAR2A (C) and JPH3 (D) are presented as means±SEMs (n=3). (E-H) Coronal brain sections of hippocampus were obtained at 1 week after S1 injection and stained using anti-NMDAR2A (E-F) and anti-JPH3 (G-H) antibodies. The insets (E-H) are high-magnification photographs of the white boxes. Images are representative of three independent experiments. Scale bars in E-H represent 300 μm and those in insets represent 50 μm. * p < 0.05 vs. control group. Con, control group; S1, S1 injected group.

Since our mRNA sequencing analysis indicated that genes involved in synaptic function may be downregulated by the S1 protein, we examined whether proteins associated with synaptic function were altered in the brains of rats injected with the S1 protein. To investigate these alterations, we performed western blot analysis on the hippocampus of S1 protein-injected rats. NMDAR2A and JPH3, key proteins for synaptic function and neural signaling, were significantly reduced 1 week after S1 injection (Fig 3B–3D). Immunohistochemistry further showed decreased NMDAR2A- and JPH3-expressing neurons in the hippocampal pyramidal layer of S1-injected rats (Fig 3E–3H). These results indicate that S1 impairs synaptic function by downregulating critical synaptic proteins, potentially contributing to cognitive deficits.

### The SARS-CoV-2 spike protein increases the protein level of HIF-1α and may suppress the expression of genes related to synaptic function

**Fig 4:**
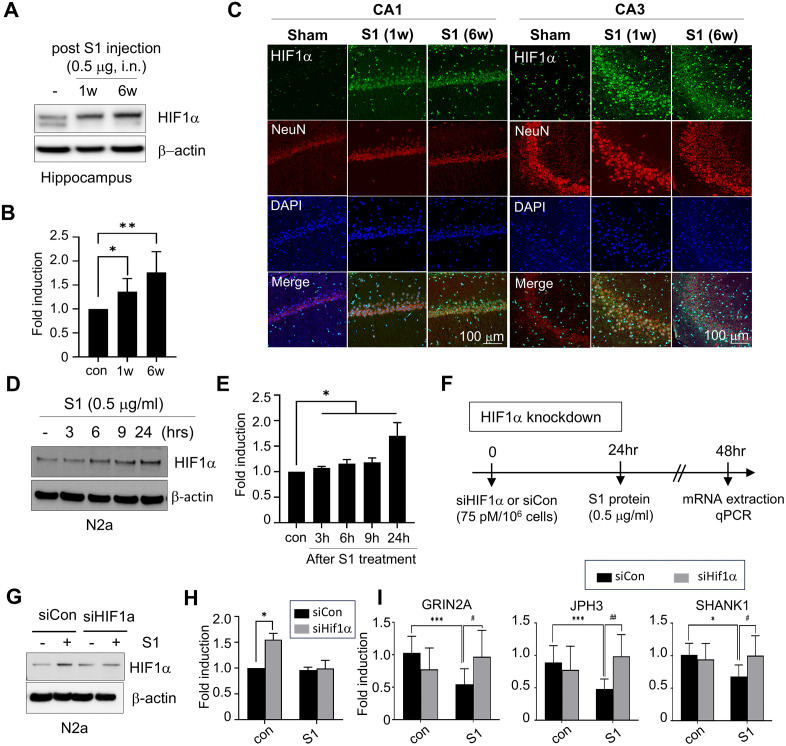
The effect of S1 to the stabilization of HIF-1α protein and gene expression related to synaptic function. (A-B) S1 (0.5 μg/ animal, intranasal) were injected and HIF-1α was assessed by immunoblotting at 1 or 6 weeks after S1 administration. Representative images are presented. The protein levels of HIF-1α at 1 or 6 weeks after S1 injection were measured by immunoblotting (A) and protein levels at each time points (B) are presented as means±SEMs (n = 3). * p < 0.05, ** p < 0.01 vs. PBS-treated control group. (C) Coronal brain sections of the hippocampus (CA1 and CA3) were obtained at 1 or 6 weeks after S1 administration and stained using anti-HIF-1α, anti-NeuN antibodies and DAPI. Photographs are representative of three independent experiments. Scale bars in C represent 100 μm. (D-E) N2a cells were incubated with S1 (0.5 μg/ mL) for 3, 6, 9, 24 hr and HIF-1α was examined by immunoblotting (D). β-actin was used as a loading control. The protein levels at each time points (E) are presented as means±SEMs (n = 3). * p < 0.05 vs. PBS-treated N2A cells. (F-I) Schematic of HIF-1α knockdown analysis in N2A cell line (F). N2a cells were transfected with murine HIF-1α siRNA (siHIF-1α, 75 pM per 10^6^ cells) or control siRNA (siCon), and then treated with S1 (0.5 μg/ mL) for 24 hr. The protein level of HIF-1α was examined using immunoblotting (G), and the quantitative data are presented as means±SEMs (n = 3) in (H). The gene expressions of GRIN2A, JPH3, and SHANK1 were assessed using a realtime PCR analysis (I). mRNA levels are presented as means ± SEMs. * p < 0.05, ** p < 0.01, ***p < 0.001 vs. control+siCon treated cells, #p < 0.05, ## p < 0.01 vs. S1 + siCon treated cells. Con, control group; S1, S1 injected group.

Hypoxia-induced activation of HIF-1α is well established to promote apoptosis and impair synaptic function. Recent studies have shown that intermittent hypoxia triggers HIF-1α-mediated redox imbalance, disrupting synaptic physiology and spatial memory [27]. Consistent with this, our NGS analysis (Fig 2) revealed that S1 alters the expression of genes associated with hypoxia, neuronal injury, and synaptic plasticity. Western blotting demonstrated a significant increase in hippocampal HIF-1α protein levels following S1 administration (Fig 4A and 4B), and immunohistochemistry confirmed increased numbers of HIF-1α-positive neurons in the CA1 and CA3 regions at both 1 and 6 weeks (Fig 4C). In vitro, S1 exposure induced a time-dependent accumulation of HIF-1α in neuronal cells, detectable as early as 6 hr and sustained up to 24 hr (Fig 4D and 4E). To investigate the functional relevance of HIF-1α stabilization, we performed siRNA-mediated knockdown of HIF-1α in N2A cells (Fig 4G and 4H). This intervention restored the expression of synaptic function-related genes, including GRIN2A, JPH3, and SHANK1 (Fig 4I), implicating HIF-1α in the suppression of synaptic gene expression and suggesting its critical role in S1-induced synaptic dysfunction.

### SARS-CoV-2 spike protein causes accumulation of p-tau and α-synuclein aggregation and may induce neuronal cell death

Emerging evidence suggests that the S1 protein may directly contribute to the accumulation of neuropathogenic proteins linked to Alzheimer’s and Parkinson’s diseases. Recent studies have shown that S1 binds to heparin and heparin-binding proteins which accelerate the aggregation of Aβ [16]. Specifically, the S1 domain interacts with α-synuclein, enhancing its aggregation and leading to mitochondrial dysfunction, oxidative stress, and cytotoxicity [18,28]. Consistent with these findings, our previous study showed that S1 protein increases α-synuclein aggregation in the substantia nigra and striatum [18]

**Fig 5:**
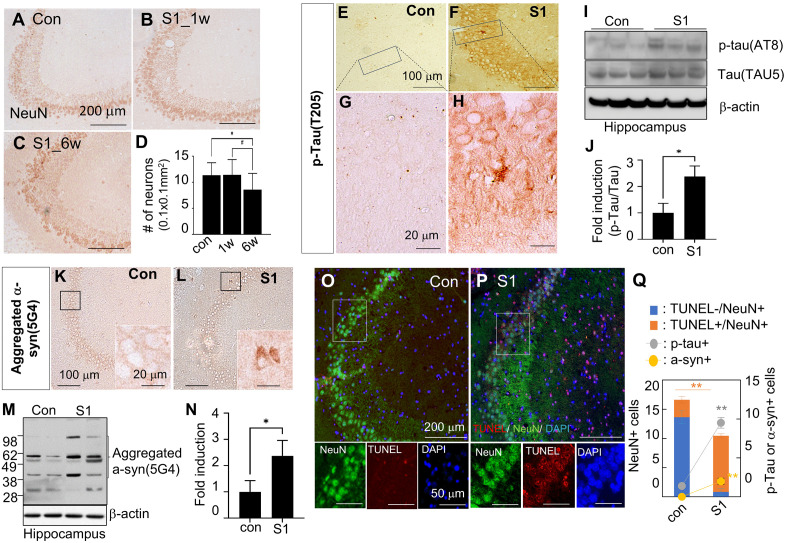
Accumulation of Alzheimer’s disease or Parkinson’s disease-related proteins and neural damage in the hippocampus following S1 administration. (A-D) Coronal sections of hippocampus (CA3) were obtained at 1 or 6 weeks after S1 injection (0.5 μg/ animal, intranasal) and stained using anti-NeuN antibody (A-C). The number of NeuN+ cells in CA3 region of hippocampus in control and S1-injected rats (6 weeks) are presented as means ± SEMs (n = 3) in (D). Photographs are representative of three independent experiments. Scale bars in A-C represent 200 μm. (E-N) Hippocampus of 6 weeks after S1 injection and control group were stained using anti-p-tau (T205) (E-H) and anti-aggregated α-synuclein (K-L) antibodies. The insets (G-H and K-L) are high magnification images of the black boxes in E-F and K-L. Photographs are representative of three independent experiments. Scale bars in E-F and K-L represent 100 μm; those in the high magnification photographs (G-H, K-L) and insets represent 20 μm. The protein levels of p-tau, total tau (I-J) and aggregated α-synuclein (M-N) were measured. Representative immunoblots and protein levels presented as means ± SEMs (n = 3). (O-Q) Brain sections of the rats at 6 weeks post S1 injection were prepared and processed for TUNEL assay and staining with anti-NeuN antibody. Representative images are shown (O-P), and the numbers of TUNEL^-^/ NeuN^+^ , TUNEL^+^/ NeuN^+^, p-tau^+^ , and α-syn ^+^ cells were counted in the CA3 region (0.1 mm^2^)(Q) and presented as means±SEMs (n = 21 from three animals). Con, control group; S1, S1 injected group.

In our study, NeuN staining revealed a significant reduction in neuronal numbers in the hippocampal CA3 region 6 weeks after S1 administration, while cortical neurons remained unaffected (Fig 5A–5D and S3 Fig). Hyperphosphorylated tau was observed in the hippocampus, and aggregated α-synuclein was detected in the hippocampus, cortex, striatum, and olfactory bulb of S1-injected rats (Fig 5E–5H, 5K, 5L and S4 Fig). Increased protein levels of p-tau (AT8) and aggregated α-synuclein was confirmed by immunoblotting (Fig 5I, 5J, 5M and 5N). TUNEL assays further revealed that 65.9% of NeuN+ cells in the CA3 region were apoptotic (Fig 5O–5Q), along with a 6.3-fold and 4.4-fold increase in p-tau+ and α-synuclein + cells, respectively. Additionally, ventricular enlargement, a marker of brain atrophy in neurodegenerative diseases [29–31], was observed in S1-injected mice at 6 weeks post-treatment (S5 Fig). These results suggest that the S1 may induce cell death linked to neurodegenerative diseases.

### Metformin mitigates the alteration of synaptic plasticity-related genes and neuropathological protein aggregation induced by SARS-CoV-2 spike protein

**Fig 6:**
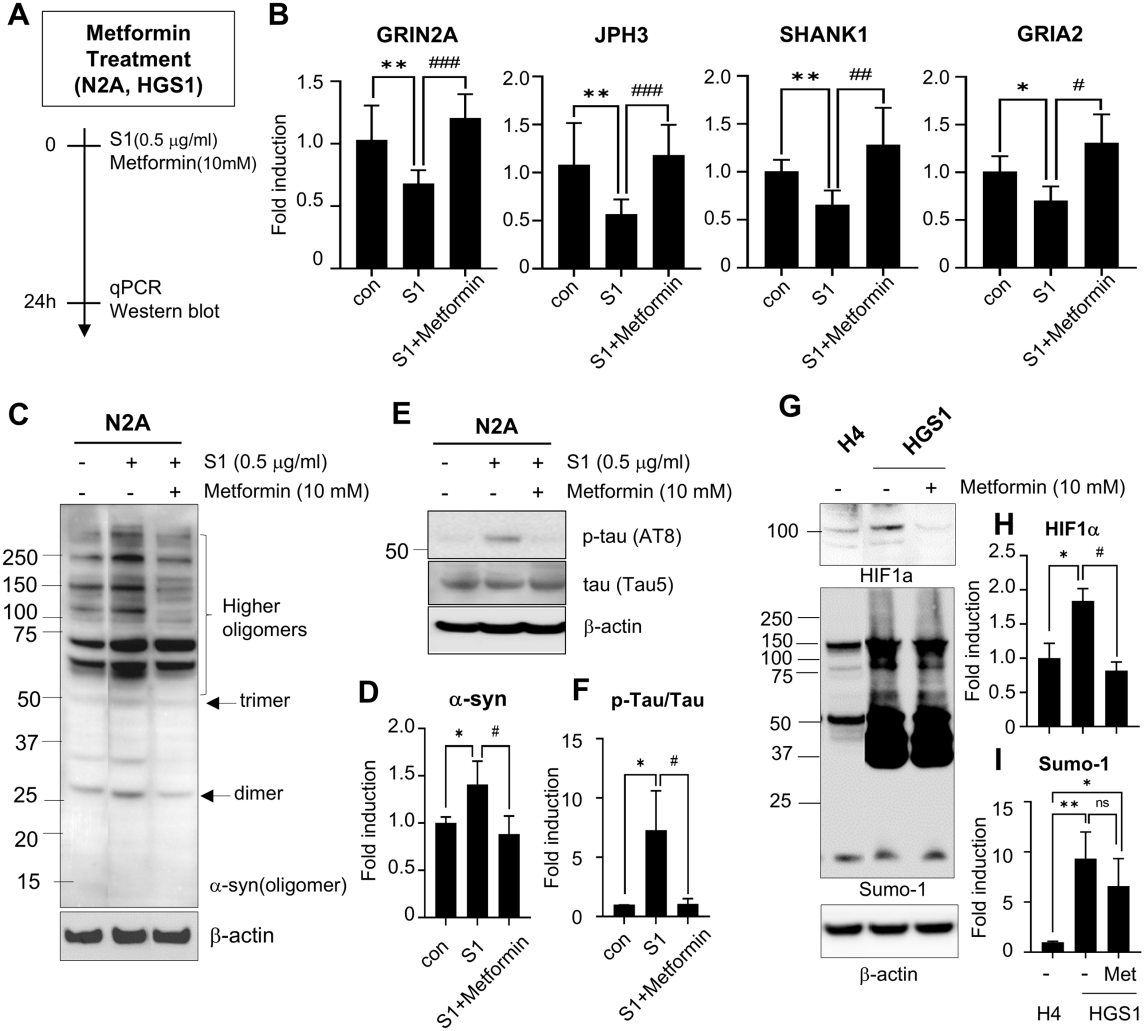
The effects of metformin to the expression of synaptic function-related genes and neuropathological protein aggregation induced by S1 protein. (A) Schematic of metformin treatment in N2A or HGS1 cell lines. (B) Effects of metformin on the expression of GRIN2A, JPH3, SHANK1, and GRIA2 in N2A cells were examined by real-time PCR after co-treatment with metformin (10 mM) and S1 (0.5 μg/ mL) for 24 hr. (C-F) Effects of metformin on the neuropathological protein aggregation were evaluated by immunoblotting after co-treatment of metformin and S1 protein for 24 hr. α-synuclein (C) and p-tau and total tau (E) were measured. The protein levels of α-synuclein (D) and p-tau/total tau (F) are presented as means±SEMs (n = 3). (G-I) Effects of metformin on HIF-1α stabilization and sumoylation were performed with H4 and HGS1 cells using immunoblotting (G). The protein levels of HIF-1α (H) and Sumo-1 (I) are presented as means±SEMs (n = 3). Representative images are shown. * p < 0.05, **p < 0.01 vs. control group, #p < 0.05, ##p < 0.01, ###p < 0.001 vs. S1 only treated group. Con, control group; S1, S1 injected group.

Metformin, a widely used antidiabetic drug, has also been reported to exert neuroprotective effects, particularly in neurodegenerative diseases such as Alzheimer’s and Parkinson’s diseases [32,33]. To assess its potential benefits in our model, we evaluated whether metformin could reverse S1-induced changes in synaptic plasticity-related genes. S1 treatment led to a marked decrease in the expression of GRIN2A, JPH3, SHANK1, and GRIA2, whereas concurrent metformin treatment restored these levels to control values (Fig 6B), suggesting a protective effect against S1-induced synaptic alterations.

Western blot analysis revealed increased α-synuclein aggregation, forming dimers and trimers, and elevated p-tau (AT8) levels in S1-treated N2A cells, both of which were significantly attenuated by metformin co-treatment (Fig 6C–6F). Given that HIF-1α stabilization can occur under normoxic conditions through oxidative stress and SUMOylation [34–36], and that SARS-CoV-2 infection enhances SUMOylation [37], we further investigated whether metformin could modulate this pathway. In H4 cells overexpressing SUMO1 (HGS1 cells), SUMO1 overexpression led to increased HIF-1α levels, which were reduced by metformin treatment (Fig 6G–6I). These findings suggest that metformin not only mitigates S1-induced alterations in synaptic gene expression but also counteracts pathological aggregation of p-tau and α-synuclein, potentially through suppression of HIF-1α SUMOylation and stabilization.

## Discussion

**Fig 7:**
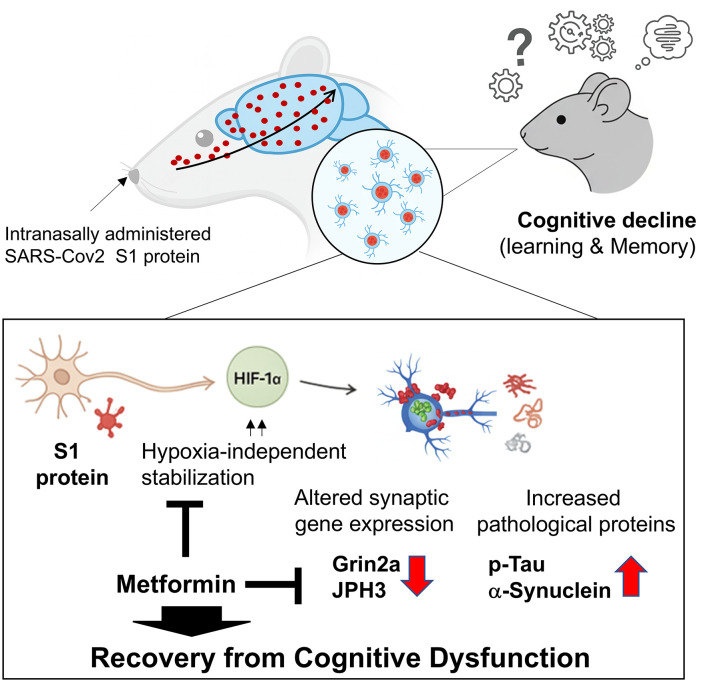
Schematic illustration of SARS-CoV-2 S1 spike protein–induced cognitive impairment and `the protective role of metformin. Intranasally administered SARS-CoV-2 S1 protein enters the rat brain and leads to cognitive decline associated with learning and memory deficits. S1 induces hypoxia-independent stabilization of HIF-1α, which alters synaptic plasticity–related gene expression. S1 exposure also promotes the accumulation of pathological proteins such as phosphorylated tau and α-synuclein through distinct pathways, collectively contributing to neuronal dysfunction. Metformin alleviates these effects by suppressing HIF-1α stabilization, thereby restoring the expression of synaptic plasticity-related genes, and by reducing the accumulation of pathological proteins, ultimately contributing to recovery from cognitive dysfunction.

Post-COVID-19 cognitive impairment, often referred to as “brain fog,” has emerged as a significant public health concern, affecting approximately 28% of COVID-19 survivors with symptoms persisting for months or even years [7,8]. Given the long-term neurological consequences of COVID-19, it is crucial to elucidate the underlying mechanisms and develop targeted therapeutic strategies In this study, we first show the molecular mechanisms of metformin as a potential treatment for post-COVID-19 cognitive decline. Our findings show that S1 protein-induced cognitive impairment follows a progressive process. Following intranasal administration, the S1 protein reaches the hippocampus, stabilizing HIF-1α and leads to a reduction in the expression of synaptic plasticity-related genes, including GRIN2A and JPH3, as early as 1 week post-administration. Additionally, S1 protein promotes the chronic accumulation of neuropathogenic factors, such as tau phosphorylation and α-synuclein aggregation, ultimately leading to neuronal cell death. Notably, we demonstrate that metformin, a widely used antidiabetic drug, effectively mitigates these pathological processes by suppressing HIF-1α stabilization and reducing neuropathogenic factor aggregation. These findings provide mechanistic insights into post-COVID-19 cognitive impairment and highlight the potential of metformin as a repurposed therapeutic agent capable of modulating key pathological cascades, including HIF-1α stabilization and protein aggregation, associated with long COVID (Fig 7).

The long-term effects of viral infections on cognitive function have been attributed to viral persistence in the central nervous system (CNS) and chronic neuroinflammation. Recent studies highlight that SARS-CoV-2 proteins can persist in various physiological processes, even after viral clearance. The SARS-CoV-2 spike protein has been detected in the skull, meninges, and cortical regions, as well as in immune cells and blood plasma for several months [12,13]. Additionally, intranasally administered S1 crosses the blood-brain barrier via adsorptive transcytosis, interacting with ACE2 in the brain and triggering neuroinflammation in the cortex, which contributes to cognitive dysfunction [14,38]. Consistent with these findings, our study confirmed rapid S1 protein entry into multiple brain regions and demonstrated that S1 exposure leads to impairments in episodic memory, spatial learning, and increased anxiety, suggesting that persistent spike protein contributes to long-term cognitive decline (Fig 1). Synaptic plasticity, which regulates the strength and efficiency of neural information transmission, is fundamental for memory formation and learning. In schizophrenia patients, reduced synaptic density and a decrease in dendritic spine numbers impair neuronal network efficiency, contributing to cognitive deficits [39]. In Alzheimer’s disease (AD), the accumulation of β-amyloid and tau proteins disrupts synaptic structures and impairs synaptic plasticity, leading to memory loss and cognitive decline [40]. In Parkinson’s disease (PD), dopaminergic neuronal loss is a key pathological feature, and interestingly, more than half of PD-related genes and risk factors are associated with synaptic dysfunction [41]. These findings suggest that disruptions in synaptic connectivity and plasticity ultimately lead to neural circuit collapse and functional loss. Our hippocampal mRNA sequencing analysis revealed that S1 significantly alters the expression of synaptic function-related genes within 1 week (Fig 2). Moreover, mRNA sequencing data from COVID-19 patients exhibited similar gene expression patterns, aligning with our findings in the animal model (S2 Fig). These results suggest that the synaptic plasticity-related gene alterations observed in both the S1 protein-injected model and COVID-19 patients may represent a pathological mechanism contributing to cognitive impairment.

HIF-1α, a transcription factor primarily induced under hypoxic conditions, is implicated in COVID-19 prognosis and neuronal metabolic regulation. Studies in non-human primates demonstrated strong HIF-1α expression in endothelial and glial cells of the perivascular regions after SARS-CoV-2 virus infection, regardless of respiratory symptoms [42]. Although generally neuroprotective, chronic HIF-1α activation impairs synaptic plasticity [27]. In sleep apnea models, elevated HIF-1α led to a 2-fold increase in ROS and severe NMDA receptor-dependent LTP impairment, while heterozygous HIF-1α knockout mice exhibited no LTP deficits [27]. In our study, persistent HIF-1α elevation in the hippocampus up to 6 weeks post-S1 administration (Fig 4) correlated with reduced synaptic plasticity-related genes, including GRIN2A and SHANK1. Importantly, siRNA-mediated knockdown of HIF-1α restored their expression, supporting a causal role of HIF-1α in S1-induced synaptic dysfunction.

Under normoxic conditions, HIF-1α typically undergoes rapid degradation via hydroxylation, acetylation, and ubiquitination by PHD, pVHL, ARD-1, and p300/CBP complexes [34]. However, we observed sustained HIF-1α stabilization after S1 exposure, suggesting a hypoxia-independent mechanism. SUMOylation, known to stabilize HIF-1α, was enhanced in SARS-CoV-2-infected cells [36,37,43]. Consistently, SUMO1 overexpression increased HIF-1α levels in our study (Fig 6), suggesting that S1 promotes SUMOylation-mediated HIF-1α stabilization under normoxic conditions. It is well established that COVID-19 induces systemic and neuroinflammation, accompanied by elevated ROS and cytokine signaling, all of which can upregulate HIF-1α [44,45]. Moreover, COVID-19-related microvascular dysfunction and tissue hypoxia have also been reported, which could further promote HIF-1α stabilization [46,47]. While our data specifically support SUMOylation as one mechanism under normoxic conditions, we acknowledge that inflammatory and hypoxia-related pathways may also converge to drive HIF-1α–mediated synaptic dysfunction in the context of SARS-CoV-2.

Metformin, the first-line treatment for type 2 diabetes, has recently attracted attention not only for its metabolic effects but also for its neuroprotective properties in various neurodegenerative diseases. Multiple epidemiological studies have consistently shown that type 2 diabetes patients receiving metformin have a lower risk of cognitive decline and dementia. For instance, long-term metformin use was associated with slower decline in global cognition and memory performance [48], reduced risk of cognitive impairment in community cohorts [49], and decreased incidence of Alzheimer’s disease in large database studies [50,51]. Beyond diabetes, several clinical studies have suggested that metformin may confer protection against post-viral complications. Notably, the COVID-OUT randomized trial compared three repurposed drugs—metformin, ivermectin, and fluvoxamine—for the prevention of long COVID in outpatients with SARS-CoV-2 infection. Among these agents, only metformin significantly reduced the incidence of long COVID (hazard ratio 0.59, 95% CI 0.39–0.89; absolute risk reduction 4.1% at 300 days), whereas ivermectin and fluvoxamine showed no benefit [52]. Consistent findings from observational studies have also reported reduced disease severity and mortality in COVID-19 patients treated with metformin [53,54]. These clinical data support the translational relevance of our findings, demonstrating that the neuroprotective actions of metformin observed in human populations align with our experimental results. In our study, metformin alleviated S1-induced synaptic dysfunction by modulating HIF-1α stabilization and reducing pathological protein aggregation, providing a mechanistic explanation for its observed clinical benefits. Collectively, these findings highlight metformin as a promising therapeutic candidate for preventing or mitigating post-COVID neurocognitive disorders.

Mechanistically, metformin activates AMP-activated protein kinase (AMPK), a key regulator of cellular metabolism that is triggered by an increased intracellular AMP:ATP ratio, and consequently suppresses mTOR signaling and NLRP3 inflammasome activation, thereby reducing neuroinflammation and oxidative stress [55]. Beyond these actions, metformin also modulates hypoxia-inducible factor-1α (HIF-1α), a central mediator of S1-induced synaptic dysfunction. By inhibiting mitochondrial complex I, metformin lowers oxygen consumption and facilitates prolyl hydroxylase–mediated hydroxylation and proteasomal degradation of HIF-1α [56]. In parallel, AMPK activation suppresses mTOR-dependent HIF-1α translation [57] and reduces ROS-driven stabilization of HIF-1α [58]. Through these converging mechanisms, metformin alleviates HIF-1α–driven suppression of synaptic plasticity–related genes and prevents dendritic collapse (Fig 6). In addition, AMPK activation enhances protein phosphatase 2A (PP2A) activity and autophagic clearance of misfolded proteins, thereby reducing tau hyperphosphorylation and α-synuclein accumulation [59,60]. Consistent with these mechanisms, our previous study demonstrated that the SARS-CoV-2 S1 protein promotes α-synuclein aggregation—a pathological hallmark of α-synucleinopathies such as Parkinson’s disease—in the substantia nigra and striatum [18]. In the present study, we further observed the accumulation of both α-synuclein and phosphorylated tau in the hippocampus of S1-injected animal models, both of which are associated with neuronal cell death (Fig 5). Importantly, metformin treatment attenuated these pathological changes, significantly reducing S1-induced α-synuclein and phosphorylated tau accumulation (Fig 6). These combined effects explain why metformin treatment reversed S1-induced deficits in synaptic plasticity and pathological protein aggregation, supporting its potential as a broadly applicable neuroprotective therapy against long COVID–related cognitive impairment.

In this study, after administering spike protein intranasally, there was a notable accumulation of tau and α-synuclein proteins, accompanied by a corresponding decline in cognitive function within the hippocampal region. These results suggest that direct delivery of spike protein to the central nervous system can induce neuropathological changes via neuroinflammation and synapse loss. Indeed, previous studies have shown that spike protein accumulation in the hippocampus is associated with increased inflammatory cytokines, and neuronal loss, leading to cognitive decline [14,61]. Nevertheless, the findings derived from these animal models cannot be unequivocally correlated with the adverse effects associated with the COVID-19 vaccination. It is established that the vaccine induces transient expression of the spike protein within the organism following intramuscular injection, with the generated protein being rapidly cleared from the system. There is limited evidence to suggest that vaccinated individuals accumulate significant levels of spike protein in the brain.

In fact, several studies suggest that the vaccination mitigates cognitive impairments associated with COVID-19 infection and does not increase the risk of psychiatric adverse events [62,63]. Consequently, the neuropathological change observed in animal studies should not be confused with complications arising post-vaccination, as the safety of the vaccination has been corroborated by a multitude of clinical and epidemiological investigations. Additional research will be required to elucidate the specific conditions under which spike protein accumulation in the hippocampus occurs and to ascertain the clinical implications of the resultant neurological alterations.

This study elucidates the molecular mechanisms by which S1 disrupts synaptic plasticity and induces neurodegenerative protein aggregation, leading to cognitive impairment. Additionally, we highlight the potential of metformin as a therapeutic intervention to mitigate these pathological changes. As the prevalence of long-term cognitive deficits following COVID-19 continues to rise, our findings provide critical insights into post-COVID neurological complications and potential treatment strategies.

## Supporting information

S1 Fig SARS-CoV-2 S1 protein alters hippocampal gene expression related to neural function, immune-related processes, and oxidative stress pathway. Heatmap of relative gene expression levels of significant Differentially Expressed Genes (DEGs) in SARS-CoV-2 S1 protein associated with neural function (A–F), immune-related processes (G and H), and regulation of superoxide metabolic process (I). These DEGs were identified based on the Gene Set Enrichment Analysis (GSEA) results shown in Fig 2.
(TIF)

S2 Fig SARS-CoV-2 S1 protein alters hippocampal gene expression in pathways relevant to COVID-19-related brain changes. (A) COVID-19-associated differentially expressed genes (DEGs) were curated from postmortem brain transcriptomic data (COVID-19 vs. age/sex-matched controls) published by Mavrikaki et al., 2022, and analyzed for enrichment of Gene Ontology (GO) biological processes. (B) Venn diagram showing the overlap between DEGs from the hippocampus of S1-injected rats and those from the brains of COVID-19 patients. Representative overlapping genes include GRIN2A and SHANK1 (see S1 Table). (C-D) (C) Schematic of gene expression profiling. (D) Gene set enrichment analysis (GSEA) of hippocampal DEGs following SARS-CoV-2 S1 protein injection, using gene sets significantly upregulated or downregulated (D) in COVID-19 patient brain. Enrichment plots demonstrate that S1-induced gene expression changes significantly recapitulate COVID-19-related transcriptional alterations, particularly in synaptic signaling and immune-related pathways.
(TIF)

S3 Fig Quantity of neurons in the cortex following SARS-CoV-2 S1 protein injection. (A-C) Representative immunohistochemical images of NeuN-positive neurons in the cortex of control rats (A) and S1-injected rats at 6 weeks post-injection (B). Insets show high-magnification views of the boxed regions, highlighting neuronal nuclei. Scale bars: 100 μm (main images), 20 μm (insets). (C) Quantification of NeuN-positive neurons within the cortex (0.1 mm² area). Data are presented as mean ± SEM (n = 12 sections from 3 animals per group). No significant difference was observed between groups.
(TIF)

S4 Fig SARS-CoV-2 S1 protein induces α-synuclein aggregation in the brain. (A-D) Representative western blot images showing aggregated α-synuclein or monomers isolated from hippocampus, cortex, striatum, and olfactory bulb of control and S1-injected rats (6 weeks after injection). Blots were probed with anti-aggregated α-synuclein antibody (5G4, upper panels) and re-probed with anti α-synuclein antibody to detect the monomer (lower panels). Red arrowheads indicate monomeric α-synuclein (~15 kDa), while arrowheads at ~38 or ~62 kDa denote higher molecular weight species corresponding to oligomeric or aggregated forms. Dashed red lines demarcate lanes from control and S1-treated groups.
(TIF)

S5 Fig SARS-CoV-2 S1 protein injection enlarges the lateral ventricles. (A–B) Representative cresyl violet-stained coronal brain sections showing the lateral ventricle in control (A) and SARS-CoV-2 S1-injected rats at 6 weeks post-injection (B). (C) Quantification of lateral ventricle area revealed significant enlargement in the S1-injected group compared to controls. Scale bar = 200 μm. Data are presented as mean ± SEM (n = 2 sections from 3 animals per group). ***p < 0.001 vs. control group.
(TIF)

S1 Table Overlapping DEGs for cognition between hippocampal transcriptomic profiles of S1-injected rats and severe COVID-19 patients. The table lists genes differentially expressed in both the hippocampus of S1-injected rats and the brains of severe COVID-19 patients (GSE188847). The direction of regulation is presented according to the S1-injected rat dataset. These overlapping DEGs are associated with cognition-related pathways (GO:0050890).
(XLSX)

S1 Raw Images Original uncropped and unadjusted Western blot images corresponding to figures presented in the main text. (PDF)

S1 Text Supplementary methods describing detailed procedures for immunohistochemistry, cresyl violet staining, and measurement of lateral ventricle size. (DOCX)
