# Supplementary figures and images for: SARS-CoV-2 spike protein causes synaptic dysfunction and p-tau and α-synuclein aggregation leading cognitive impairment: The protective role of metformin
Source: PLoS One. 2025 Nov 7;20(11):e0336015. doi: 10.1371/journal.pone.0336015 (PMC12594341; doi:10.1371/journal.pone.0336015)

Fig 1

A

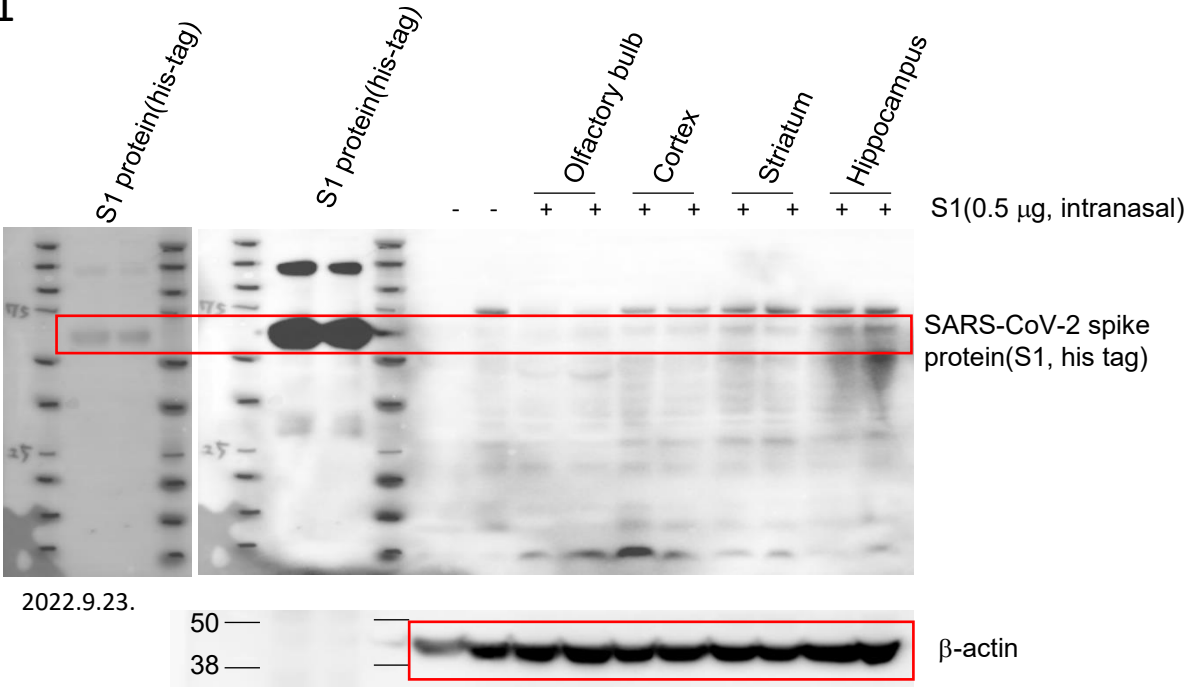

Fig 3

B

2022.12.10.

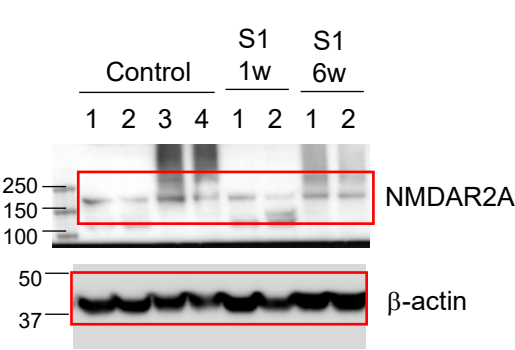

2023.2.7.

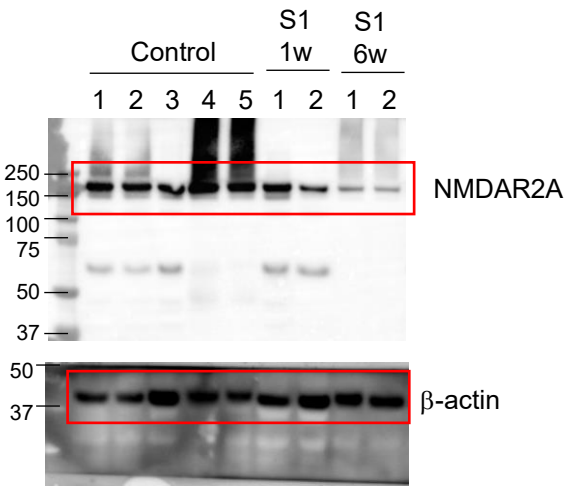

2023.9.15.

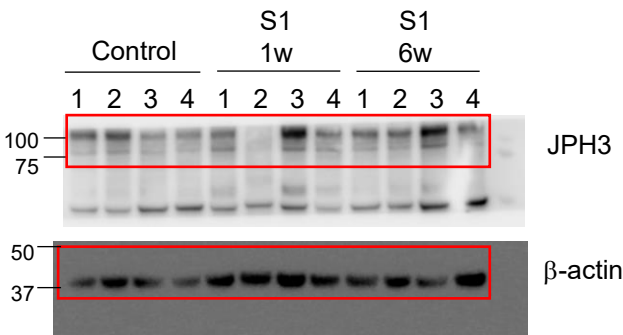

Fig 4

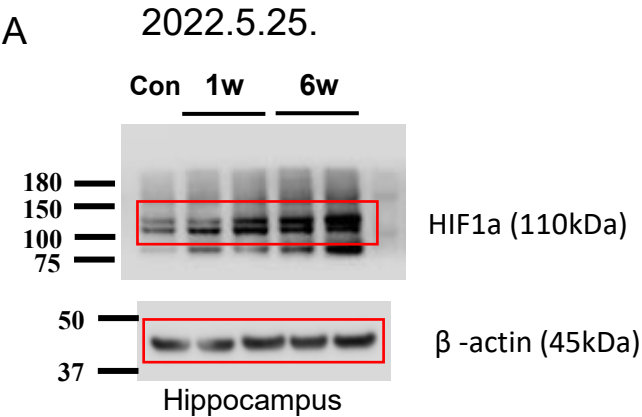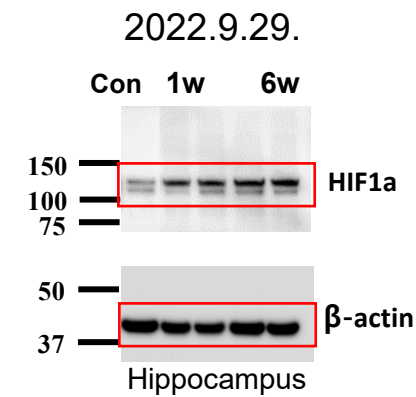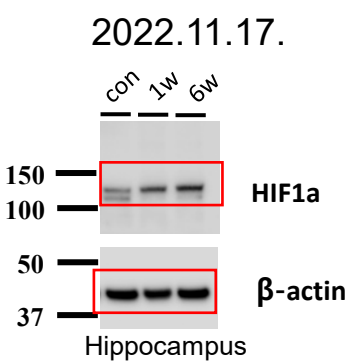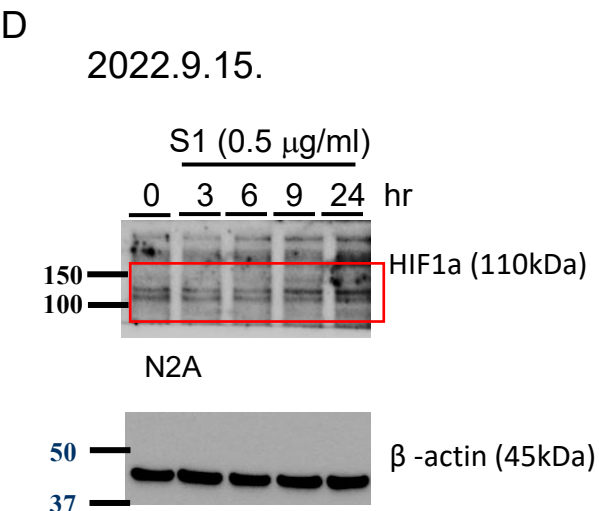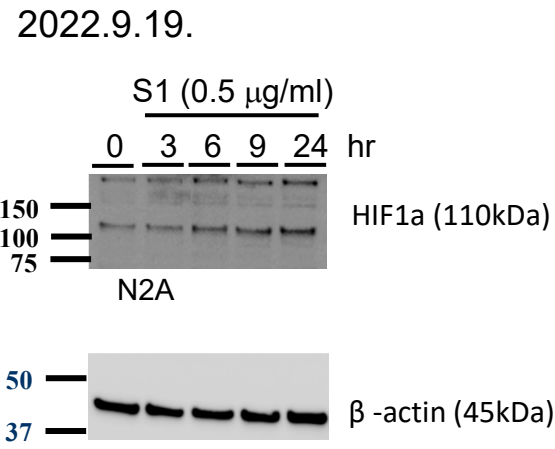

Fig 4

E      2023.1.13.

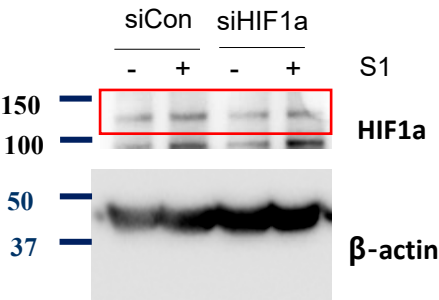

2023.1.17.

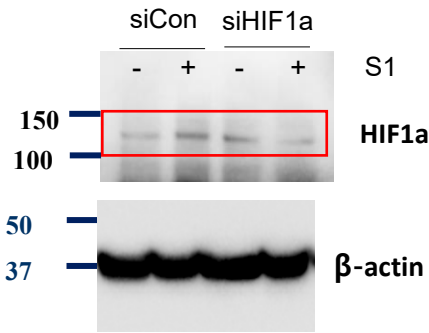

2023.1.21.

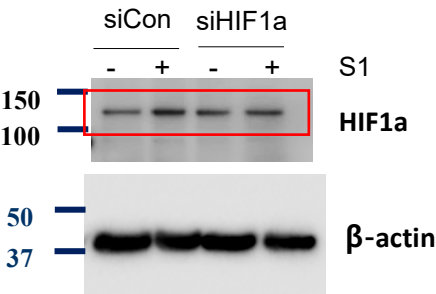

Fig 5

I

2024.6.21

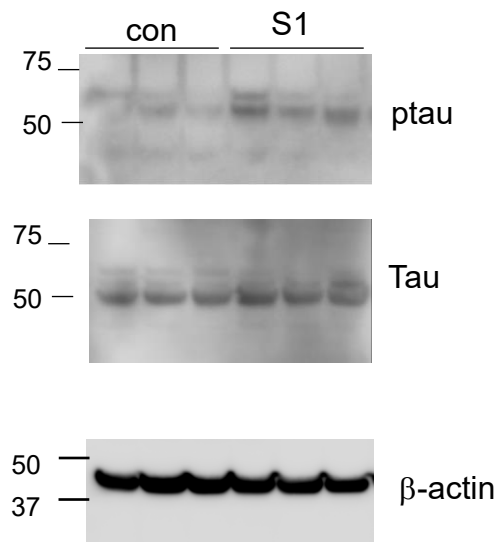

M

2023.8.16.

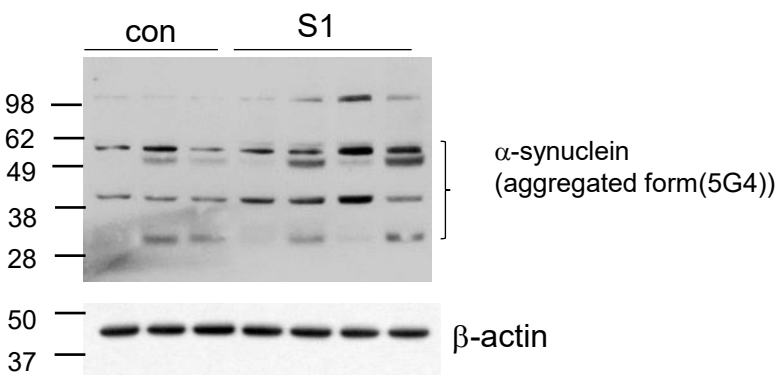

2023.5.23.

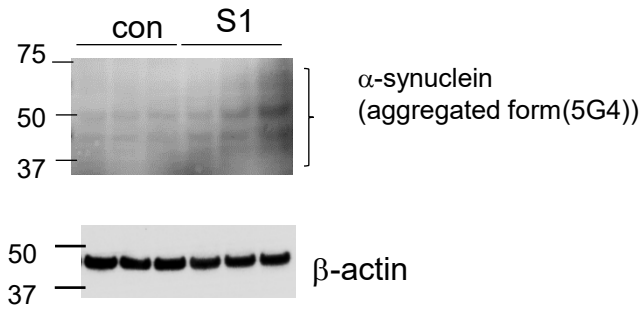

Fig 6

C

2024.5.1.

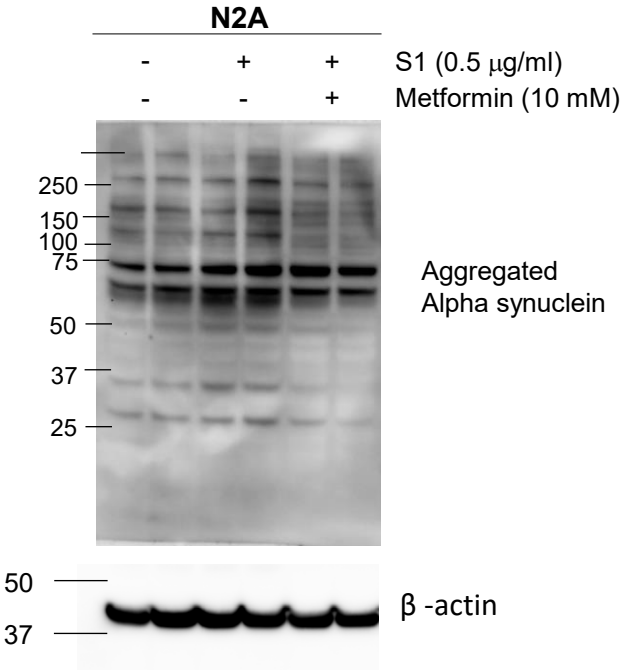

E

2024.7.5.

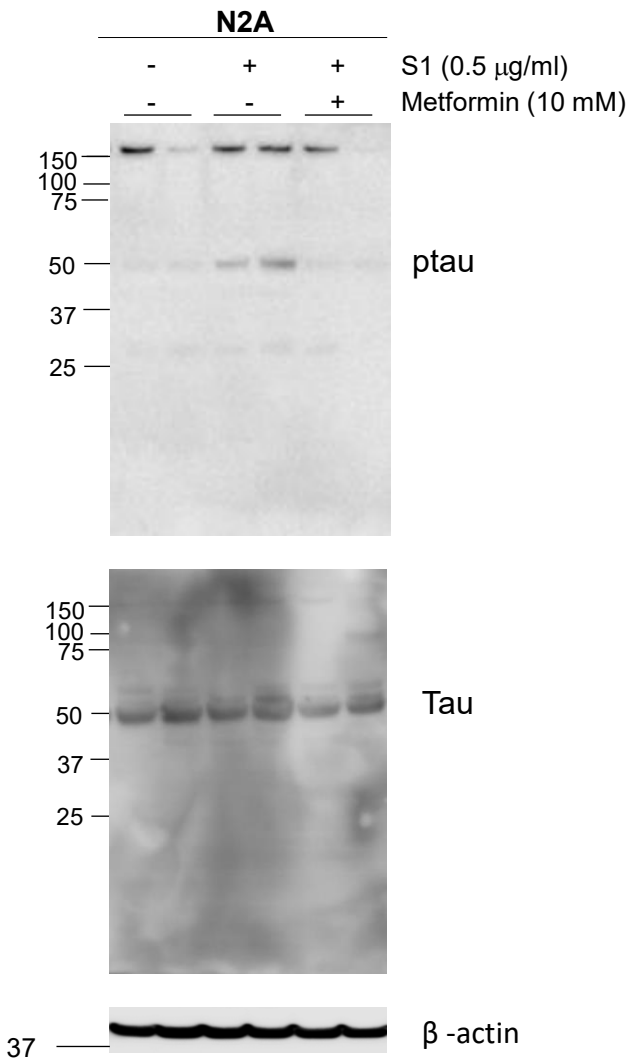

Fig 6

G

2024.5.1.

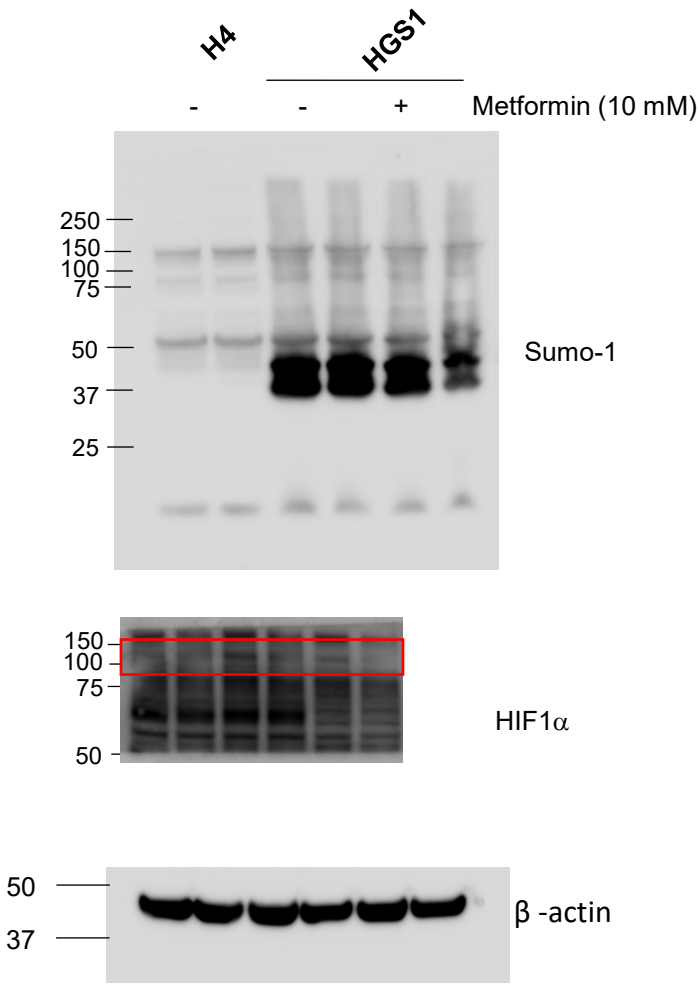

Supplement: S1 Raw Images — (PDF) [file pone.0336015.s007.pdf]
