# Supplementary material for: SARS-CoV-2 spike protein causes synaptic dysfunction and p-tau and α-synuclein aggregation leading cognitive impairment: The protective role of metformin
Source: PLoS One. 2025 Nov 7;20(11):e0336015. doi: 10.1371/journal.pone.0336015 (PMC12594341; doi:10.1371/journal.pone.0336015)
Supplement: S1 Text — (DOCX) [file pone.0336015.s008.docx]

**Supplementary information**

SARS-CoV-2 spike protein causes synaptic dysfunction and p-tau and α-synuclein aggregation leading cognitive impairment: The protective role of metformin

Hye-Kyung Lee^1^, Ji Young Choi^1^, Jung Hyun Park^1^, Moon Han Chang^1^, Jung Ho Park^2^ and Young Ho Koh^1*^

^1^Division of Brain Diseases Research, Department of Chronic Disease Convergence Research, Korea National Institute of Health, 187 Osongsaengmyeong2(i)-ro, Osong-eup, Heungdeok-gu, Cheongju-si 28159, Republic of Korea

^2^ Nexmos Inc., U-Tower, 767, Sinsu-ro, Suji-gu, Yongin-si, Gyeonggi-do, 16827, Republic of Korea

*Corresponding author:

E-mail: kohyoungho122@gmail.com (YHK)

**Supplementary materials and methods**

**Immunohistochemistry**

Immunological staining of brain sections was performed using paraffin-embedded tissue sections. Primary antibodies were diluted as follows: anti-NeuN (Millipore, Burlington, MA) was used at a concentration of 1:100. Experiments were repeated at least three times, and representative images are presented.

**Cresyl violet staining**

Brains were embedded in paraffine and 5 μm-thick sections were prepared. The sections were deparaffinized in zylene and rehydrated through graded series of ethanol and cresyl violet staining was performed. In brief, the sections were incubated in 0.1–0.5% cresyl violet acetate solution (Abcam, Cambridge, UK) for 5–10 min at room temperature. Excess stain was removed by briefly rinsing in distilled water, followed by differentiation in 95% ethanol, dehydration in absolute ethanol, and clearing in xylene. Slides were then coverslipped using a mounting medium.

**Measurement of lateral ventricle size**

For staining of lateral ventricle, the brain sections (bregma 0.2 mm, interaural 9.2 mm) were stained using cresyl violet actate solution. Lateral ventricle size was assessed by measuring the stained ventricular area using ImageJ software (NIH, Bethesda, MD, USA).
